# Aluminum overload in the reverse osmosis dialysis era: does it exist?

**DOI:** 10.1080/0886022X.2022.2104165

**Published:** 2022-10-03

**Authors:** Mei-Yin Chen, Shih-Hsiang Ou, Nai-Ching Chen, Chun-Hao Yin, Chien-Liang Chen

**Affiliations:** a Department of Nutrition and Food Service, Kaohsiung Veterans General Hospital, Kaohsiung, Taiwan; b Department of Nursing, Shu-Zen Junior College of Medicine and Management, Kaohsiung, Taiwan; c Division of Nephrology, Kaohsiung Veterans General Hospital, Kaohsiung, Taiwan; d Faculty of Medicine, School of Medicine, National Yang Ming Chiao Tung University, Taiwan; e Department of Neurology, Kaohsiung Chang Gung Memorial Hospital and Chang Gung University College of Medicine, Kaohsiung, Taiwan; f Division of Education and Research, Kaohsiung Veterans General Hospital, Kaohsiung, Taiwan; g Institutes of Clinical Medicine, National Yang Ming Chiao Tung University, Taiwan; h Institution of Precision Medicine, National Sun Yat-sen University, Kaohsiung, Taiwan

**Keywords:** Aluminum, aluminum overload, desferroxime, dialysis

## Abstract

**Background:**

Aluminum accumulation is a well-described complication in dialysis patients. Improvements in hemodialysis technology have possibly eliminated the occurrence of aluminum overload. Limited evidence suggests that aluminum overload may decline in the era of aluminum removal from dialysis fluids, even with the use of aluminum binders.

**Methods:**

We examined the data from January 2014 to June 1, 2020, identified through our electronic records, to evaluate the desferrioxamine (DFO) test results for aluminum overload. The presentation and treatment of aluminum overload were recorded.

**Results:**

Ninety-nine dialysis patients were enrolled for the DFO test. Forty-seven patients (47.5%) were identified as DFO test positive for aluminum overload, of which 14 (14/47) patients had symptoms, including one patient with an unexplained fracture, eight patients with unexplained anemia despite high-dose erythropoiesis-stimulating agents, and five patients with hypercalcemia (serum calcium >11 mg dL^-1^). None of the patients with aluminum overload developed encephalopathy. Only four of the 47 patients had microcytic anemia. Patients requiring longer treatments (>10 months versus <10 months) had similar basal serum aluminum (*p* = 0.219) but had an increase in serum aluminum after DFO (*p* = 0.041). Furthermore, the treatments decreased erythropoietin doses in the aluminum overload group, with serum total alkaline phosphatase levels <60 U L^-1^ (*p* = 0.028).

**Conclusion:**

We concluded that aluminum overload existed in the reverse osmosis dialysis era. In light of non-obvious symptoms, such as anemia and bone turnover change, serum aluminum in dialysis patients should be monitored in countries using aluminum-based phosphate binders, despite reverse osmosis dialysis.

## Introduction

Patients with chronic kidney disease are at a high risk of aluminum overload due to poor renal excretion of aluminum [1]. It has been suggested that dialysis centers should serially follow serum aluminum levels as a screening tool for patients at risk of aluminum toxicity [2–4]. Aluminum can be eliminated from the dialysate using reverse osmosis techniques. Improvements in hemodialysis technology and patient care may have eliminated the occurrence of aluminum overload. The low prevalence of aluminum toxicity among dialysis patients has raised a debate about the need to continue screening for aluminum overload in dialysis patients [5,6], which the Kidney Disease Outcomes Quality Initiative (K/DOQI) guidelines suggest routinely [2]. Aluminum-containing phosphate binders are an important source of aluminum exposure in dialysis patients. Although aluminum-based phosphate binders used as short-term therapy are considered by the K/DOQI guidelines, some patients with hyperphosphatemia under calcium-based phosphate binders complicated by hypercalcemia still extend the duration of aluminum therapy. The Dialysis Outcomes and Practice Patterns Survey 2011 report found 12.7% and 13.7% usage of aluminum binders in Spain and Australia, respectively, but 0%, 0.1%, and 0.3% in Belgium, the United States, and Japan, respectively [7]. Sevelamer hydrochloride and lanthanum carbonate have been suggested as non-calcium and non-aluminum phosphate binders, respectively. However, these newly developed medications have not become popular because of their high costs. Some experts believe that aluminum-based phosphate binders continue to play a role in clinical nephrology practice [8]. Indeed, there are several other possible sources of aluminum besides phosphate binders because aluminum salts are used in water treatment, bakery products, and pharmacy processes. Aluminum containers, utensils, and cookware may cause aluminum to migrate from the instrument into the solution [9–11]. Therefore, aluminum overload in the reverse osmosis dialysis era remains controversial.

Excess aluminum may cause dialysis dementia, erythropoietin-resistant microcytic anemia, or bone disease [12]. Patients with aluminum-related bone diseases may experience muscle weakness, musculoskeletal pain, or fractures. Aluminum overload and other complications can develop in patients on dialysis, especially during parathyroidectomy (PTX) [13]. Patients may have hypercalcemia due to aluminum blocking the additional calcium uptake into the bone from the additional calcium in the calcium phosphate binder with intensive vitamin D treatment or the high calcium content dialysis fluid superimposed by a decrease in bone turnover during anti-resorption therapy or hypoparathyroidism after PTX [12,14,15]. An early diagnosis of aluminum toxicity is important to ensure effective therapy. Bone biopsy specimens are considered the gold standard for diagnosing aluminum overload in dialysis patients [2,15,16]. In the absence of a bone biopsy, diagnosis can be made by measuring serum aluminum levels before and after a noninvasive deferoxamine (DFO) test to identify subjects with an increased body burden of aluminum [2,17–19]. DFO tests should be performed if there are elevated serum aluminum levels (60 − 200 µg L^-1^) or clinical signs and symptoms of aluminum toxicity, or before PTX if the patient has been exposed to aluminum for at least four months [2].

The improvements in hemodialysis technology and restrictions on the use of aluminum-based phosphate binders have resulted in a low prevalence of aluminum toxicity (2%) among hemodialysis patients in the United States [4–6]. However, there is 25% of aluminum bone diseases [20], 16.2% of aluminum overload in patients at an Iran dialysis center [21], and 8% of aluminum overload in Taiwan [22]. The diversity of the aluminum reports may be due to the different country circumstances, dialysis care and definition of aluminum overload in bone biopsy, single serum aluminum or a positive DFO test.

The clinical presentation and incidence of aluminum overload in the reverse osmosis dialysis era with the permitted use of aluminum-based phosphate binders remain unknown. We examined data from the Kaohsiung Veterans General Hospital dialysis clinic to evaluate serum aluminum levels and the DFO test for aluminum overload. The incidence, clinical presentation, and treatment of aluminum overload were also analyzed.

## Materials and methods

### Design

This retrospective study evaluated noninvasive test strategies for predicting aluminum overload in a group of dialysis patients. There is a standard treatment protocol for hyperphosphatemia in dialysis patients. Most of the patients are prescribed calcium-based phosphate binders, but when serum phosphate levels exceed 6.0 mg dL^-1^ or the product of serum calcium x serum phosphate exceed 55 mg^2^ dL^-2^, aluminum hydroxide 324 mg tablets are prescribed 1# to 4# three times a day. These test parameters were evaluated against the pathological diagnosis of aluminum overload based on the deferoxamine test (‘gold standard’). The study was conducted in accordance with the Declaration of Helsinki and the protocol was approved by the institutional review board of the hospital (IRB No: KSVGH21-CT4-03). DFO tests were performed for clinical reasons to determine the level of aluminum toxicity in patients. All patients who started dialysis in January 2014 and were still on treatment on June 1, 2020, were identified through hospital medical records. The Kaohsiung Veterans General Hospital has routinely provided a high-flux dialyzer for outpatient dialysis patients since 2002. All hemodialysis patients underwent three-weekly high-flux dialysis, using a high-flux dialyzer membrane such as Polysulfone (Asahi Kasei Medical Co., Ltd., Japan) and Toraysulfone (Toray Industries, Inc., Japan) in the dialysis center. These patients underwent DFO tests for 48 h. Briefly, 5 mg kg^-1^ DFO was administered intravenously at the end of dialysis. Aluminum concentrations were measured pre-DFO and 48 h post-dose (pre-dialysis). During and after the DFO test, the hemodialysis staff was asked to report any adverse events.

Patients with a clinical suspicion of aluminum toxicity or elevated serum levels of intact parathyroid hormone (iPTH) (>800 pg mL^-1^), indicating the need for PTX, were included. Aluminum toxicity included symptoms of general bone pain or proximal muscle weakness, easy fracture, progressive cognitive dysfunction, unexplained hypercalcemia, and anemia without iron deficiency under high-dose erythropoietin. According to the K/DOQI guidelines, a DFO test should be performed if there are suspected clinical signs and symptoms of aluminum toxicity or before parathyroid surgery if the patient has been exposed to aluminum [2]. A positive DFO test (an increase of ≥50 μg L^-1^ in serum aluminum concentrations post-DFO [5 mg/kg] administration)) was used to diagnose aluminum overload [2].

### DFO treatment

The goal of treatment was to alleviate the symptoms of aluminum overload. End-stage renal disease with aluminum overload was administered at a standard dose (5 mg.kg^-1^). Two weeks before the study, patients avoided aluminum-containing medications as much as possible. DFO was administered once a week at a dose (5 mg kg week^-1^) according to the K/DOQI guidelines. Biochemical and hematological parameters and adverse events due to DFO were also recorded.

### Laboratory methods

Aluminum-free glassware and tubes were used in the study. They were maintained in 20% (v/v) nitric acid overnight and subsequently washed seven times with deionized water prior to use. All reagents were of analytical grade. Ultrapure water (>18MΩ˙cm) used throughout this analytical procedure was prepared using a deionized water system (Milli-Q, Millipore). Stock solutions (1000 mg.L^-1^) of aluminum (III), suprapure nitric acid, and Triton-X 100 were purchased from Merck Taiwan-Sigma-Aldrich. The serum aluminum concentrations were measured in the laboratory at Kaohsiung Medical University affiliated Hospital using an A800 atomic absorption spectrophotometer (PerkinElmer, Norwalk, CT, USA) after suitable dilution with 4% nitric acid (containing 0.1% Triton) solution [23,24]. To verify the contamination of storing or preparing samples in the study, ultrapure saline solutions were used as blank samples. The aluminum levels in blank samples were found to be below the limit of quantification (2.2 ng L^-1^), which means that contamination of aluminum from glass devices or tubes can be ignored under this analytical method. Quality control was performed strictly using standard reference materials with intra-assay of coefficients of variation (ClinChek® Serum Control Level with a coefficient of variation of 4.6% and Seronorm Trace Elements Serum Control level 2 with a coefficient of variation of 5.0%). To ensure that patients were not exposed to aluminum-contaminated water and dialysate during HD, we collected at least two dialysate samples from the inlet and outlet of the dialysate port of the dialyzer every year to confirm that they met the criteria of the Association for the Advancement of Medical Instrumentation water treatment equipment. Ten ml of blood was collected from the vascular access of patients undergoing dialysis.

### Statistical analysis

Continuous variables were checked for normal distributions using the Kolmogorov–Smirnov *Z* test and compared using Student’s *t*-test or paired *t*-test, if appropriate. All continuous variables are expressed as the mean ± standard deviation. Ordinal demographic data were analyzed using the *χ*^2^ test or Fisher’s exact test. The strength of correlation was obtained with the Pearson’s correlation coefficient. Major statistical analyses were performed using Statistical Package for the Social Sciences version 15.0 (SPSS Inc., Chicago, IL, USA). Statistical significance was set at *p* < 0.05.

## Results

Altogether, 99 patients were enrolled in the analysis, of which 47 patients were DFO-positive (47.5%). Fifty-four patients (iPTH:1088.9 ± 573.3 pg mL^−1^) surveyed DFO tests because of severe hyperparathyroidism (history of iPTH >800 pg mL^−1^) and were candidates for PTX. The remaining 45 patients (iPTH:154.6 ± 191.6 pg mL^−1^) had DFO due to unexplained anemia (15 patients), relatively low bone turnover (29 patients), and unexplained multiple rib fracture (one patient). The clinical and demographic characteristics of the participants are shown in Tables 1 and 2, respectively.

**Table 1.** Baseline characteristics of dialysis patients—consisted of two groups according to serum aluminum (<20 [μg L^-1^] or ≥20 [μg L^-1^]).

	Total group	Aluminum <20	Aluminum ≥20	*p* Value
	(*n* = 99)	(*n* = 33)	(*n* = 66)	
Sex-male	54 (55%)	19 (58%)	35 (53%)	0.669
Age (year)	54.0 ± 13.2	54.1 ± 12.5	53.6 ± 14.5	0.840
Duration of dialysis(month)	49.3 ± 31.0	49.7 ± 22.3	49.1 ± 34.7	0.926
Hemodialysis/peritoneal dialysis	92 (92.9)	32 (97.0)	60 (90.9)	0.267
Hematocrit (%)	30.3 ± 5.6	30.7 ± 4.6	30.1 ± 6.0	0.655
Calcium (mg dL^-1^)	9.5 ± 1.1	9.6 ± 1.0	9.4 ± 1.1	0.573
Phosphate (mg dL^-1^)	5.7 ± 1.7	5.6 ± 1.9	5.7 ± 1.6	0.774
Alkaline phosphatase (U L^-1^)	95.2 ± 72.0	91.0 ± 49.4	97.3 ± 80.8	0.695
Intact parathyroid hormone (pg mL^-1^) *	669.4 ± 643.4	857.4 ± 678.8	578.3 ± 610.1	0.043
Serum aluminum (μg L^-1^) **	33.1 ± 25.3	11.7 ± 5.3	43.7 ± 24.6	<0.001
Serum aluminum after DFO (μg L^-1^) **	86.0 ± 53.9	35.1 ± 21.2	111.5 ± 46.8	<0.001
ΔAluminum (μg L^-1^) **	53.0 ± 3.5	23.5 ± 16.9	67.7 ± 29.8	<0.001
Percentage of DFO positive**	47	1/33	46/66	<0.001
**Underlying disease (%)**				
CGN	28	10	18	
CIN	26	11	15	
DM	19	4	15	
IgA nephropathy	3	0	3	
Nephrosclerosis	8	1	7	
Lupus	3	0	3	
Others	12	5	7	

DFO: desferroxime; CGN: chronic glomerulonephritis; CIN: chronic interstitial nephritis; DM: diabetes mellitus.

*<0.05; **<0.01.

**Table 2.** Baseline characteristics of dialysis patients—consisted of two groups according to deferoxamine (DFO) positive or negative.

	DFO negative	DFO positive	*p* Value
	(*n* = 52)	(*n* = 47)	
Sex-male/female	29/23	25/22	0.797
Age (year)	54.0 ± 13.2	54.1 ± 12.5	0.451
Duration of dialysis(month)	44.7 ± 22.0	54.4 ± 38.2	0.122
Hemodialysis/peritoneal dialysis	47 /5	45 /2	0.299
Hematocrit (%)	30.1 ± 5.3	30.5 ± 5.9	0.747
Mean corpuscular volume, fL	90.6 ± 8.9	89.3 ± 6.2	0.413
Calcium (mg dL^-1^)	9.6 ± 1.1	9.4 ± 1.1	0.314
Phosphate (mg dL^-1^)	5.5 ± 1.6	5.9 ± 1.8	0.186
Alkaline phosphatase (U L^-1^)	89.5 ± 50.5	101.3 ± 89.2	0.425
Intact parathyroid hormone (pg mL^-1^)	772.4 ± 708.6	557.7 ± 550.2	0.099
Serum aluminum (μg L^-1^) **	18.6 ± 11.8	49.0 ± 26.7	<0.001
Serum aluminum after DFO (μg L^-1^) **	46.9 ± 25.2	129.4 ± 42.8	<0.001
ΔAluminum (μg L^-1^) **	28.3 ± 16.0	80.3 ± 25.5	<0.001
**Underlying disease (%)**			
CGN	17	11	
CIN	14	12	
DM	7	12	
IgA nephropathy	1	2	
Nephrosclerosis	5	3	
Lupus	2	1	
Others	6	6	

Data are expressed as mean ± standard deviation.

CGN: chronic glomerulonephritis; CIN: chronic interstitial nephritis; DM: diabetes mellitus.

*<0.05; **<0.01.

In this study, the prevalence of the risk of aluminum overload (serum aluminum >20 µg L^−1^) was 69.7%. There were 22 patients with serum aluminum levels between 20 and 30 µg L^−1^, 17 with serum aluminum levels between 30 and 40 µg L^−1^, and 6 patients with serum aluminum levels between 40 and 50 µg L^−1^. The serum aluminum level was higher than 60 µg L^−1^ in 10 patients. The study demonstrated a positive correlation between △serum aluminum, after infusion of a standard dose of DFO and dialysis duration (*r* = 0.673, *p* < 0.001). The 10 patients with serum aluminum levels higher than 60 µg L^−1^ had a trend with a relatively longer dialysis duration (65.4 ± 45.9 vs. 47.5 ± 28.7 months) to induce aluminum accumulation (*p* = 0.08).

As shown in Table 1, we further used the K/DOQI guidelines of serum aluminum 20 μg L^−1^ to analyze the clinical status. However, 1 out of 33 (3.3%) patients had aluminum levels <20 μg L^−1^. Meanwhile, the 46/66 patient group had an aluminum overload of >20 μg L^−1^. Between the two groups, there were significant differences between the serum aluminum levels, increase in aluminum levels, positive rates of the DFO test (*p* < 0.001), and relatively lower intact parathyroid hormone levels (*p* = 0.043). Patients with serum aluminum levels >20 μg L^−1^ vs. those with serum aluminum levels <20 μg L^−1^ showed no significant differences in age, sex, and history of diabetes mellitus.

**Figure 1.**
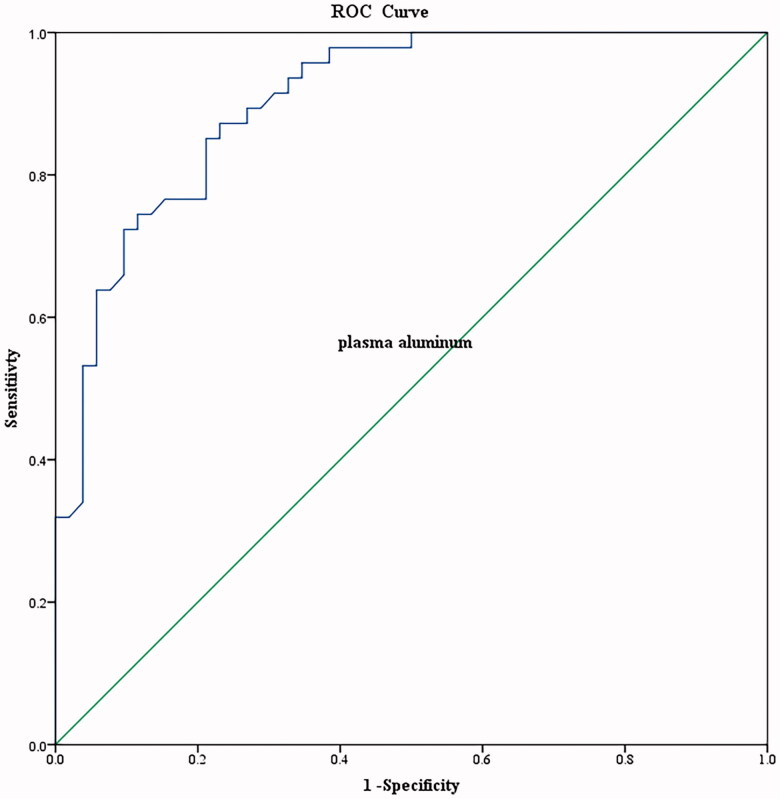
Receiver operating characteristic curve (ROC) analysis for discrimination of aluminum overload group and aluminum without overload group. The ROC curve is a necessary tool for better interpretation of the results of serum aluminum classification studies. Notably, the ROC curves are empirical curves for sensitivity and specificity spaces.

The baseline characteristics of the dialysis patients, consisting of two groups according to DFO positivity or negativity, are summarized in Table 2. There were significant differences in serum aluminum levels, aluminum levels after the DFO test, and the increment in aluminum levels after the DFO test (*p* < 0.001). As shown in Tables 1 and 2, serum aluminum levels were strongly and significantly associated with an increased risk of aluminum overload (*p* < 0.01). Therefore, serum aluminum level could be a predictor of aluminum overload. An increase in serum aluminum level by10 µg. L^−1^ was associated with an approximately 200% increase in the odds of aluminum overload (Table 3). Using serum aluminum predicted aluminum overload (area under curve: 0.904) with a cutoff level of 24 μg. L^-1^, the sensitivity and specificity were 87.2 % and 76.9%, respectively in this study (Figure 1) (*p* < 0.01).

**Table 3.** Relative odds of aluminum overload by quartiles of random aluminum levels.

Variable	OR (95% Confidence interval)	*p* Value
Baseline aluminum (AL) < 20 μg L^-1^	Ref	–
20 ≤ Baseline AL <30	29.09 (3.33 − 253.96)	0.002
30 ≤ Baseline AL <40	58.67 (6.34 − 542.91)	<0.001
40 ≤ Baseline AL <50	160.00 (8.56 − 2989.45)	0.001
50 ≤ Baseline AL	320.00 (27.31 − 3762.85)	<0.001

Aluminum toxicity includes the following signs and symptoms: acute dementia, osteomalacia with bone pain, multiple non-healing fractures, unexplained anemia [12], and hypercalcemia occurring in these patients when attempts are made to treat hyperparathyroidism by administering calcium and 1,25(OH)_2_D [14]. None of the patients with aluminum overload showed overt symptoms of dementia. There were 14 (14/47) patients with symptoms that included bone symptoms in one patient with an unexplained fracture, unexplained anemia in eight patients requiring high-dose erythropoietin (excluding hyperparathyroidism) and hematological symptoms, and unexplained hypercalcemia in five patients (serum calcium **≥** 11 mg dL^-1^) with relatively low bone turnover syndrome. The remaining 33 (33/47) patients had no overt symptoms. The mean corpuscular volume (MCV) of aluminum overload was approximately 89.3 ± 6.2 fL Only 4 of the 47 patients had microcytic anemia.

Four patients refused DFO treatment. Only 43 patients underwent the DFO treatment. After treatment without parathyroidectomy, the rib fractures and hypercalcemia improved. Furthermore, there was a decrease in the frequency of blood transfusion and cessation of blood transfusion in the three patients. Eighteen patients required at least 10 months of aluminum overload treatment. Twenty-five patients required less than 10 months of DFO treatment. As shown in Table 4, longer DFO treatments were especially effective in patients with a higher increase in serum aluminum after DFO (*p* = 0.041), but similar basal serum aluminum levels (*p* = 0.219). After DFO treatment, there was a significantly larger MCV (*p* = 0.001), decreased incidence of microcytic anemia (*p* = 0.007), and a trend of increased hematocrit (*p* = 0.076) (Table 5). As shown in Table 6, DFO treatment decreased erythropoietin dosage, especially in patients with lower serum total alkaline phosphatase levels (<60 µ L^-1^) (*p* = 0.028).

**Table 4.** Serum basal aluminum levels and the difference in serum aluminum levels by the standard deferoxamine (DFO) test at the standard dose (5 mg/kg) are necessary for ≥10 months.

	<10 months of DFO successful treatment, medical treatment (*n* = 25)	≥10 months of medical treatment (*n* = 18)	*p* Value
Serum basal aluminum level (μg L^-1^)	50.4 ± 30.4	49.5 ± 23.5	0.913
Serum aluminum level (μg L^-1^) after DFO	125.2 ± 43.1	141.91 ± 43.5	0.219
Δaluminum* (μg L^-1^) after DFO	74.8 ± 19.1	92.4 ± 30.8	0.041

*<0.05; **<0.01.

**Table 5.** Biochemistry data using the standard deferoxamine (DFO) test at the standard dose (5 mg.kg^-1^) for 6 months.

Parameter	Before DFO treatment (*n* = 43)	After DFO treatment (*n* = 43)	*p* Value
Serum calcium (mg.dL^-1^)	9.4 ± 0.2	9.3 ± 0.2	0.404
Serum phosphorus (mg.dL^-1^)	6.1 ± 0.3	5.8 ± 0.2	0.225
Alkaline phosphatase (U.L^-1^)	97.9 ± 11.6	102.9 ± 10.9	0.413
intact parathyroid hormone (pg.mL^-1^)	503.7 ± 66.7	572.7 ± 76.4	0.281
Hematocrit (%)	31.3 ± 0.8	32.4 ± 0.8	0.076
Mean corpuscular volume**, fL	89.0 ± 1.0	91.0 ± 1.1	0.001
Mean corpuscular volum*e* < 80*, fL	4/39	2/41	0.007
Serum Aluminum** (μg.L^-1^)	48.0 ± 23.2	37.9 ± 17.6	0.004
△serum aluminum** (μg.L^-1^) after DFO	82.5 ± 30.8	55.4 ± 34.5	<0.001

*<0.05; **<0.01.

**Table 6.** Comparison of differences in biochemical and hematological parameters in the standard dose (5 mg kg^-1^) DFO treatment group for 6 months.

Characteristics	Al*p* ≤ 60(*n* = 13) Before	After	*p* Value	Al*p* > 60(*n* = 30) Before	After	*p* Value
Aluminum (μg.L^-1^)	32.7 ± 3.7	35.4 ± 14.0	0.594	58.2 ± 44.0	39.0 ± 19.2	0.061
△aluminum* (μg.L^-1^) after DFO	90.4 ± 40.0	66.1 ± 33.0	0.038	83.6 ± 27.3	51.1 ± 35.0	0.001
Calcium (mg.dL^-1^)	9.4 ± 0.8	9.4 ± 1.0	0.848	9.3 ± 1.1	9.2 ± 1.1	0.672
Phosphate (mg.dL^-1^)	6.6 ± 1.4	6.2 ± 1.4	0.506	5.8 ± 1.9	5.5 ± 1.7	0.222
Intact parathyroid hormone (pg.mL^-1^)	434.5 ± 447.2	431.4 ± 405.1	0.422	620.2 ± 604.6	751.5 ± 657.4	0.125
Hematocrit (%)	30.8 ± 4.4	31.8 ± 4.8	0.484	30.4 ± 6.6	31.2 ± 6.3	0.364
Mean corpuscular volume*, fL	90.8 ± 5.4	92.8 ± 5.9	0.077	88.1 ± 6.6	89.8 ± 7.2	0.004
Erythropoietin dose changes* (unit)/month	25179.5 ± 8505.6	22538.5 ± 38778.7	0.028	23691.4 ± 11607.1	22135.8 ± 11468.4	0.269
Ferritin	513.4 ± 260.7	474.1 ± 265.4	0.485	585.4 ± 397.0	587.5 ± 371.1	0.973

ALP: Alkaline Phosphatase.

*<0.05; **<0.01.

The K/DOQI guidelines recommend DFO for the treatment of patients on dialysis with aluminum overload. However, DFO has side effects such as itchy skin, nausea, myalgia, shock, and mucormycosis [25]. In this study, no severe overt side effects were reported at 5 mg kg^-1^per week DFO test or treatment.

## Discussion

The DFO test is a noninvasive method for identifying patients with aluminum overload. Aluminum overload was observed in 3.3% of dialysis patients with aluminum <20 μg L^−1^; even 69.7% of patients with levels above the threshold had an aluminum overload. The prevalence of patients who were DFO positive and compatible with aluminum overload in our study was up to 47.5%. None of the patients had neurological symptoms, but 14 (14/47) had symptoms of aluminum overload in the hematological and bone systems. Furthermore, a longer treatment course of aluminum overload was associated with a higher increase in serum aluminum levels in the DFO test (*p* = 0.041), with similar baseline serum aluminum levels.

The Kaohsiung Veterans General Hospital uses reverse osmosis as a medical facility using water. After reverse osmosis, water enters the dialysis water system and encounters another reverse osmosis system and deionization instruments. In other words, the dialysis water undergoes reverse osmosis twice. Using reverse osmosis and deionization instruments, the water is considered dialysate-qualified. The water meets the criteria of the AAMI water treatment equipment for hemodialysis dialysate. The improvements in hemodialysis technology and the use of aluminum-based phosphate binders have resulted in a low prevalence of 2% aluminum toxicity among hemodialysis patients in the United States, Japan, and other countries [4–8]. Our findings identified 47 patients (47.5%) as DFO test positive for aluminum overload. These results contradict previous findings of a low prevalence of 2% aluminum toxicity in the United States, but are consistent with 8%, 16.2%, and 25% of aluminum overload where the use of aluminum-based phosphate binders is permitted (Taiwan, Iran, Brazil) [20–22]. Indeed, there are several other possible sources, such as bakery products and aluminum containers. However, the prevalence of using aluminum phosphate binders in different countries is consistent with the difference according to the Dialysis Outcomes and Practice Patterns Survey, which accounts for only 0.1% of aluminum phosphate binders in the United States dialysis centers [7]. Furthermore, the different results between relatively contemporaneous groups of patients may reflect referral patterns, such as unexplained anemia, bone fracture, and inappropriate bone turnover due to parathyroid hormone. Based on our findings, we believe that this problem may occur in many countries that have not limited the use of aluminum-based phosphate binders because of a lack of awareness [26].

Can single serum aluminum levels provide an indirect estimation of bone aluminum content? We found that patients with higher aluminum levels were more likely to have aluminum overload than those with aluminum levels <20 μg L^-1^ (Table 1), consistent with reports of an association between increased serum aluminum levels and the incidence of aluminum overload [2–4,27–30]. We attempted to define the value of performing random tests to evaluate the levels of aluminum and found that this test performs very well as a screen for aluminum overload (Tables 3 and 4 and Figure 1). Serum aluminum predicted aluminum overload, AUC = 0.904, with a cutoff level of 24 μg L^−1^; the sensitivity and specificity were 87.2% and 76.9%, respectively. In clinical practice, deferoxamine (DFO) test should be performed if there are elevated serum aluminum levels (60–200 µg L^−1^) or clinical signs and symptoms of aluminum toxicity [2]. However, the clinical symptoms of aluminum overload are not overt. We suggest the use of serum aluminum levels of 20 µg L^−1^ to screen for aluminum overload because patients with serum aluminum levels <20 μg L^-1^ had only 3.3% of aluminum overload. Following clinical practice with serum aluminum levels (60–200 µg L^−1^) may delay the treatment of patients with aluminum overload [2].

As shown in Table 4, patients showing a higher △serum aluminum after infusion of a standard dose of DFO required a longer duration of DFO treatment (>10 months), whereas those showing a lower △serum aluminum tended to require <10 months of treatment (*p* < 0.05). However, there was no difference in basal serum aluminum levels in the two groups with a differing duration of treatment. Our study is compatible with a previous study with similar basal aluminum levels but different DFO test reports in patients who ingested aluminum hydroxide and calcium carbonate [31]. Furthermore, there was one study to point out that baseline plasma aluminum concentrations had lower sensitivity to predict aluminum-related osteodystrophy (sensitivity, 43%), whereas the DFO test was found more sensitive (sensitivity 94%) to predict aluminum-related osteodystrophy [18]. This means that △serum aluminum, after infusion of a standard dose of DFO, provides a more accurate estimation of the aluminum content of the tissue and bone.

In patients with hyperparathyroidism, the parathyroid hormone can protect against aluminum deposition in the bone. PTX in patients with chronic renal failure is associated with increased aluminum deposition on the bone surface, possibly as a result of low bone formation [13]. The protection decreases in patients who have undergone PTX or anti-resorption therapy or during vitamin D treatment for hyperparathyroidism, as these treatments could decrease bone turnover, worsen lower turnover symptoms [32–34] and hypercalcemic osteomalacia [14]. Considering the current case of secondary hyperparathyroidism, necessary parathyroidectomy and possible anti-resorption therapy may lower bone turnover. Therefore, it is generally suggested that aluminum bone disease should be excluded before lowering bone turnover treatment, such as PTX and bone resorption therapy. Hence, early screening and diagnosis of aluminum overload are important before lowering bone turnover treatments.

What were the clinical symptoms of aluminum overload in this study? Aluminum toxicity includes the following signs and symptoms: (1) acute dementia, (2) osteomalacia with bone pain, multiple non-healing fractures, particularly the ribs, (3) hypercalcemia encountered in patients when medications are administered to treat hyperparathyroidism by calcium and 1,25(OH)_2_D, and (4) unexplained anemia. None of the patients with aluminum overload showed overt symptoms of dementia. Most of the patients were asymptomatic. Only 14 (14/47) had aluminum overload symptoms in the hematological and bone systems. The symptoms improved after aluminum chelation treatment. DFO treatment improved the mean corpuscular volume of red blood cells (*p* = 0.001) (Table 5). Previous reports have shown that aluminum overload is associated with microcytic anemia [12,35,36]. However, only four (4/47) of the patients with aluminum overload showed microcytic anemia. Most patients (43/47) with aluminum overload did not show microcytic anemia, which is consistent with other studies that showed no microcytosis in patients with modest degrees of aluminum overload [37,38]. Furthermore, DFO treatment could decrease erythropoietin doses for anemia treatment, especially in patients with lower bone turnover, such as those with serum total alkaline phosphatase levels (< 60 U L^−1^) (Table 6). The side effects of DFO are dose-dependent, and common side effects have been noted with doses of 20 − 40 mg kg^-1^ of body weight [39–41]. The DFO dose used in this study was 5 mg kg^-1^ per week, and there were no overt side effects during treatment. We believe that most of these side effects are mild and are neglected by patients and staff.

This study has some limitations. First, we used a 5 mg kg^-1^ dose of DFO to test for aluminum overload. Bone biopsy is considered the gold standard method for diagnosing aluminum overload in patients undergoing dialysis. Second, this study enrolled patients undergoing dialysis. The number of study participants was relatively small, even when serum aluminum and biochemistry were checked in the same laboratory. Additional prospective longitudinal investigations with larger numbers of patients are necessary.

We conclude that although aluminum overload exists, it might be easily ignored in the reverse osmosis dialysis era. Aluminum overload was not associated with any neurological symptoms in this study. In light of the non-obvious symptoms of aluminum overload, such as normocytic anemia and bone turnover change, serum aluminum in dialysis patients should be monitored in countries with substantial usage of aluminum-based phosphate binders, despite the era of reverse osmosis dialysis. Clinicians must be aware of random aluminum levels as a test for aluminum overload and should consider the DFO test if necessary.

## Data Availability

The data generated during the current study are available from the corresponding author upon reasonable request.
